# Enabling Diagnostic Resulting as a New Category of Secondary Genomic Findings

**DOI:** 10.3390/jpm12020158

**Published:** 2022-01-26

**Authors:** Michael F. Murray

**Affiliations:** Yale Center for Genomic Health, Yale School of Medicine, New Haven, CT 06510, USA; michael.murray@yale.edu

**Keywords:** genomic medicine, secondary findings, health screening, DNA-based screening, diagnostic result, healthcare system, diagnostic process, molecular diagnosis, precision medicine

## Abstract

Over the past decade, the secondary analysis of existing DNA datasets for clinical resulting has become an established practice. However, this established practice is typically limited to only one category of secondary genomic findings, the identification of “disease risk”. Diagnostic resulting has been left out of secondary genomic findings. In medical practice, diagnostic resulting is triggered when a test is ordered for a patient based on a recognizable clinical indication for evaluation; most genetic and genomic testing is carried out in support of diagnostic evaluations. The secondary analysis of existing DNA data has the potential to cost less and have more rapid turnaround times for *diagnostic results* compared to current DNA diagnostic approaches that typically generate a new dataset with every test ordered. Worldwide, innovative health systems could position themselves to deliver valid secondary genomic finding results in both the established category of *disease risk results*, as well as a new category of *diagnostic results*. To support the ongoing delivery of both categories of secondary findings, health systems will need comprehensive genomic datasets for patients and secure workflows that allow for repeated access to that data for on-demand secondary analysis.

## 1. Introduction

The process of reporting secondary genomic findings is now well established within many testing laboratories. These clinical results are designated secondary findings because they are derived from existing DNA datasets [1]. Currently, the total number of existing DNA datasets are limited and using them as the source of secondary findings is referred to as “opportunistic”, that is to say it is based on the recognition of an opportunity that has arisen independently [2]. Significant effort has focused on optimizing the reporting of secondary findings in those circumstances where DNA datasets have been created for an unrelated primary intent. If we wish to realize the full potential of genomic medicine, then the process of reporting secondary genomic findings needs to: (1) transition from opportunistic to deliberate datasets and (2) add clinically indicated *diagnostic results* as secondary findings alongside the currently established practice of returning *disease risk results*.

## 2. Transitioning Secondary Findings Approaches from Opportunistic to Deliberate

Approximately a decade ago, a conversation began in earnest regarding the potential clinical value of existing DNA sequence data for patient care beyond its initial intended use [3]. The discussion was prompted by the successful clinical implementation of comprehensive DNA sequencing (genome and exome) as a diagnostic tool in the setting of rare disease [4]. The DNA datasets being created for these diagnostic tests not only have the potential to contain important genomic risk information unrelated to the rare disease question at hand, but in most cases, they are the only readily available opportunity to uncover these important *disease risk results* for these patients and their families [3].

The American College of Medical Genetics and Genomics (ACMG) has helped prompt systematic approaches to the screening of existing DNA datasets from clinical exome and genome sequencing through its guidance on secondary findings [1,5]. This approach to the DNA-based screening of existing diagnostic datasets has been a widely accepted component of the standard approach for many clinical laboratories and the healthcare providers who order diagnostic exome or genome testing.

Distinct from clinical exome and genome sequencing in rare disease diagnosis, another successful example of delivering secondary findings builds on opportunistic DNA datasets created in the context of healthcare-system-associated research cohorts. In this example, large projects such as Geisinger’s MyCode used non-CLIA research exomes as the source of the available dataset [6]. This approach required an appropriate consenting process as well as research result confirmations within a clinical laboratory in order to identify secondary findings of genomic risk [6]. These clinical findings are then delivered to patients and their care providers through the healthcare system’s electronic health record.

In the two established use cases for secondary genomic findings described here, the primary intent for the creation of DNA datasets was either rare disease diagnosis or human subject research. Once created, these DNA datasets were then made available for an intentional interrogation for secondary findings related to genomic risk. Implementing genomic risk screening through these opportunities has been foundational to gaining insight into the potential benefits associated with the clinical delivery of secondary genomic findings. Building on the knowledge and experience from opportunistic use cases, new strategic pathways can be built to serve a far larger percentage of the population through the intentional creation of DNA datasets. Deliberately created datasets with the primary intent of supporting all aspects of an individual’s healthcare can be generated. The secondary findings associated with deliberately created DNA datasets would not need to be limited to genomic risk screening but could also be made accessible for routine and repeated probing in order to report clinically indicated secondary genomic findings.

## 3. Expanding Secondary Findings Strategies to Include *Diagnostic Results*

A diagnosis is the culmination of a process set in motion by a clinical indication, such as a sign or a symptom. In contrast, the established practice of delivering genomic risk screening results as secondary findings occurs independently of any clinical indication. In fact, screening specifically seeks to identify risk in those who would otherwise go unrecognized as there is no identified health need [7]. Risk screening proceeds at a different pace than the diagnostic process.

Recent work from the Institute of Medicine highlights the potential for diagnostic delays to contribute to patient harm [8], thus making a case for timeliness in the availability of diagnostic testing results. This seems likely to become an increasingly important issue in clinical genomics as DNA-based testing applications broaden to involve more types of practitioners and more use cases, and expectations grow for fast turnaround times on genomic results that guide therapeutic or surgical decision making (e.g., pharmacogenomics and cancer syndrome management) [9,10].

Currently, when a genetic test is requested, it often takes two or more weeks for clinical laboratories to: receive a patient’s DNA sample, sequence it, analyze the data, and render a clinical result report. If an individual’s DNA dataset was generated and held securely in a clinically compliant manner prior to the clinical indication, the turnaround-time from test request to diagnostic result would be significantly decreased. With deliberately acquired preemptive DNA datasets, the fulfillment of a test order would be limited to data analysis and reporting. A diagnostic genomic test result generated in this manner would be analogous to the practice of generating diagnostic imaging test results from secondary reads of existing MRI or CT scan datasets rather than carrying out a new scan; in both scenarios, time and resources are saved by using existing data [11]. Preemptive DNA datasets could be made available for both types of secondary genomic findings, namely *disease risk results* as well as rapid *diagnostic results* in response to a clinical indication.

The hope exists that an individual’s genome can someday be used to preemptively map out all health risks. However, any attempt at an all-inclusive genomic risk resulting approach that precludes any need for diagnostic resulting currently faces two discrete limitations [7]. These limitations are: insufficient data for comprehensive DNA variant interpretation and inadequate understanding of how to predict individualized clinical outcomes associated with pathogenic DNA variants. It is noteworthy that the vast majority of the human DNA variants that have been identified fall into the clinical classification of VUS (or variants of unknown significance). It is also important to note that there is no validated process for predicting an individualized risk of disease penetrance and age of onset even when a person has a known pathogenic variant associated with some of the best-studied gene–disease pairs [7]. While progress in addressing both of these obstacles is being made, they will most likely remain as obstacles to comprehensive genomic risk prediction for decades to come. Limitations in our capacity to carry out comprehensive risk screening will leave us in need of diagnostic resulting for many years to come.

The types of clinical indications that might prompt secondary diagnostic findings include: (1) unanticipated acute medical problems and (2) results associated with an uncertain screening value in healthy individuals but a diagnostic value in the setting of disease. Examples of unanticipated acute health issues include individual genetic susceptibility related to infection and trauma [12,13], and examples of results where there is anticipated value in the setting of the diagnostic process include problems such as renal failure or hemochromatosis [14,15,16,17]. A specific example of variants where the screening value, but not the diagnostic value, has been debated for over 20 years are the well-known disease-associated variants of the *HFE* gene (i.e., H63D and C282Y) [15,16,17]. In the diagnostic context, a system that is optimized for clinically indicated secondary findings could rapidly generate *HFE* gene clinical results. These examples give context to the call to enable diagnostic resulting (alongside disease risk resulting) as a new category of secondary genomic findings. The number of additional diagnostic tests that could be run would depend on the specifics of the dataset, but the turnaround times from order to result for items on the test menu could potentially be very rapid compared to current strategies.

## 4. Conclusions

A future can be imagined where each of us have a securely held comprehensive genomic dataset available for the generation of both *disease risk results* and *diagnostic results*. This would mean routine genomic risk screening independent of demonstrated clinical needs, as well as rapid on-demand results when a patient demonstrates the need for diagnostic testing. This model could potentially offer a robust strategy that avoids repetitive sample collection for new data creation every time a genetic result is sought in an individual’s health care, by having a high-quality dataset that is able to be queried and is available within the healthcare delivery system. Functioning programmatic examples of this comprehensive approach to secondary genomic findings do not currently exist; however, many elements of this type of system have been established.

Over the last decade, individual U.S.-based health systems have carried out innovative efforts to advance genomic medicine, and they have funded it through combinations of internal institutional support, philanthropic gifts, and commercial collaborations [6,18,19]. Real-world evidence for the model of genomic medicine proposed here could now be developed within innovation-driven health systems anywhere in the world. Such pilot programs would provide opportunities to develop models for best practice. For health systems seeking to initiate pilot programs using this proposed approach, the key unanswered questions include: what is the frequency with which clinically useful queries of pre-emptive datasets will occur? Additionally, can this model for genomic medicine be financially supported and sustained more broadly?

## Data Availability

Not applicable.
